# A Comparative Analysis of Early Ventilator Mechanics in COVID-19 vs. Non-COVID-19 ARDS: A Single-Center ED-Based Cohort Study

**DOI:** 10.3390/healthcare13172139

**Published:** 2025-08-27

**Authors:** Murtaza Kaya, Ceyda Nur Irk, Mehmed Ulu, Harun Yildirim, Mehmet Toprak, Sami Eksert

**Affiliations:** 1 Department of Emergency Medicine, Faculty of Medicine, Kutahya Health Sciences University, Kutahya 43100, Turkey; harun.yildirim@ksbu.edu.tr; 2 Department of Cardiology, Faculty of Medicine, Kutahya Health Sciences University, Kutahya 43100, Turkey; ceydanur.irk@ksbu.edu.tr; 3 Department of Emergency Medicine, Adiyaman University, Training and Research Hospital, Adiyaman 02040, Turkey; mehmed.ulu@ksbu.edu.tr; 4 Department of Emergency Medicine, Kutahya City Hospital, Kutahya 43100, Turkey; mehmet.toprak@ksbu.edu.tr; 5 Department of Anesthesiology and Reanimation, Faculty of Medicine, Health Sciences University, Gulhane Training and Research Hospital, Ankara 06010, Turkey; sami.eksert@sbu.edu.tr

**Keywords:** acute respiratory distress syndrome, COVID-19, respiratory mechanics, mechanical ventilation, emergency service, hospital, severity of illness index, Charlson comorbidity index

## Abstract

**Background and Aim**: Mechanical ventilatory support is often required in patients with acute respiratory distress syndrome (ARDS). However, early differences in ventilatory mechanics and severity scores between COVID-19 and non-COVID-19 ARDS patients remain unclear. This study aimed to compare respiratory parameters and clinical severity scores in COVID-19 and non-COVID-19 ARDS patients managed in the emergency department (ED) and evaluate their association with in-hospital mortality. **Methods:** In this retrospective cohort study, adult patients with ARDS (PaO_2_/FiO_2_ < 300 mmHg) who received mechanical ventilation in the ED were included. Ventilator parameters and clinical severity scores (SOFA, APACHE II, PSI, and Charlson Comorbidity Index) were recorded at the 120th minute after intubation. Patients were categorized as COVID-19 or non-COVID-19 ARDS, and outcomes were compared between survivors and non-survivors. Logistic regression was used to identify independent predictors of in-hospital mortality. **Results:** A total of 70 patients were enrolled (32 COVID-19, 38 non-COVID). Plateau pressure, driving pressure, and PEEP were significantly higher in COVID-19 patients, while compliance was without statistical significance. Overall, in-hospital mortality did not differ significantly between the COVID-19 (53.1%) and non-COVID-19 groups (71.1%, *p* = 0.12). Mechanical power (21.6 vs. 16.8 J/min, *p* = 0.01) and Charlson Comorbidity Index (6 vs. 5.5, *p* = 0.02) were significantly higher in non-survivors across the full cohort. Among clinical scores, SOFA was significantly higher in the COVID-19 group (*p* = 0.02), and APACHE II was significantly higher in non-survivors within the COVID-19 subgroup (*p* = 0.02). In multivariate analysis, mechanical power and Charlson Comorbidity Index were associated with mortality. **Conclusions:** COVID-19 patients with ARDS exhibited higher early ventilatory pressures than non-COVID-19 patients, yet early respiratory mechanics were not independently associated with mortality. Mechanical power and Charlson Comorbidity Index were significantly associated with in-hospital mortality. These findings underscore the need to consider both ventilatory load and systemic health status in early outcome assessments of ARDS patients.

## 1. Introduction

Mechanical ventilation (MV) is a cornerstone of supportive care in patients with acute respiratory failure and acute respiratory distress syndrome (ARDS) [1]. The COVID-19 pandemic significantly increased the demand for MV, with a substantial proportion of patients progressing to severe hypoxemia and requiring invasive support [2]. While COVID-19-associated ARDS shares some clinical features with classical ARDS, accumulating evidence suggests that it may represent a distinct phenotype with different respiratory mechanics. These differences include preserved lung compliance despite severe hypoxemia, altered plateau pressures, and unique responses to positive end-expiratory pressure (PEEP). These variations have raised important questions about the applicability of standard ARDS ventilatory strategies to COVID-19 patients [3]. The results of recent physiologic investigations, such as the matched-cohort study by Grieco et al., have demonstrated that although COVID-19 patients may present with slightly higher compliance and ventilatory ratios, overall mechanical behavior and recruitability remain largely comparable to ARDS from other causes, especially in early phases of mechanical ventilation [4].

In the emergency department (ED), where many patients receive initial ventilatory support, understanding the physiological differences between COVID-19 and non-COVID-19 ARDS is crucial for guiding early ventilator settings and improving outcomes. Most comparative studies on ventilatory mechanics, however, have been conducted in ICU populations, with limited data from the ED setting [5]. Thus, there is a significant knowledge gap regarding early ventilatory management and clinical outcomes, specifically in the ED context, making this study particularly important. Moreover, the impact of ventilator parameters—such as tidal volume, driving pressure, and compliance—on clinical outcomes, particularly mortality, remains inadequately examined in COVID-19 versus non-COVID-19 patients during the initial resuscitation phase.

We aimed to compare the mechanical ventilation profiles and associated mortality of COVID-19-positive and -negative patients who required invasive ventilation in the emergency department, thereby contributing additional clinical data on early ventilatory management in acute care.

## 2. Materials and Methods

### 2.1. Study Design and Setting

This retrospective cohort study was conducted between 1 March 2022 and 1 March 2023 in the emergency department (ED) and the affiliated pandemic intensive care unit (ICU) of a tertiary academic hospital. During the peak of the pandemic, this ICU functioned as a critical care unit until patients could be transferred to other intensive care units. The ICU is structurally and administratively affiliated with the emergency department and is primarily managed by emergency medicine specialists to ensure consistent ventilatory care. The corresponding author served as the primary physician responsible for this unit and holds certification in mechanical ventilation management. Ethical approval for the study was obtained from the local Non-Interventional Clinical Research Ethics Committee (Approval No: 2022/02-08; Date: 9 February 2022).

### 2.2. Patient Selection

As this was a retrospective study, no formal sample size calculation was performed. Adult patients (≥18 years) who received invasive mechanical ventilatory support in the emergency department with a preliminary diagnosis of COVID-19–associated acute respiratory distress syndrome (ARDS) were eligible for inclusion. ARDS was defined according to the Berlin criteria as a PaO_2_/FiO_2_ (P/F) ratio below 300 mmHg at the initiation of respiratory support. Patients were subsequently followed in the emergency department and its affiliated pandemic intensive care unit (ICU). Those with no international travel history, no known exposure, and two consecutive negative PCR tests on alternate days were classified as the non-COVID-19 group. Patients who were directly admitted from the emergency department to other intensive care units were not included in the study.

### 2.3. Data Collection

Patient data were collected, reviewed, and verified by an emergency medicine specialist. These data included baseline demographic characteristics (age, sex, and comorbidities), as well as clinical severity scores: Sequential Organ Failure Assessment (SOFA), Acute Physiology and Chronic Health Evaluation II (APACHE II), and Pneumonia Severity Index (PSI). To assess comorbidity burden, the Charlson Comorbidity Index (CCI) was calculated using the MedCalc online calculator. Ventilator parameters were recorded at the 120th minute after achieving optimal mechanical ventilation settings, including appropriate FiO_2_ and PEEP levels. The recorded parameters included static compliance (Crs), driving pressure (ΔPrs), PaO_2_/FiO_2_ ratio, peak inspiratory pressure (Ppeak), plateau pressure (Pplat), tidal volume (TV), and positive end-expiratory pressure (PEEP). In addition, mechanical power (MP) was calculated to evaluate the intensity of ventilation. MP values were calculated using the patients’ recorded ventilator parameters from the Excel data sheet, according to the simplified Gattinoni-derived formula proposed by Giosa et al. [6].

MP = 0.098 × TV (L) × RR × (Ppeak − 0.5 × (Pplat − PEEP))

All ventilator data were obtained using a Biyovent R ventilator (PN: 6515-7315-0003, Aselsan, Yenimahalle, Turkey; production date: August/2020).

### 2.4. Ventilation and Sedation Strategy

Patients were initially managed in the awake prone position as tolerated, with non-invasive mechanical ventilation (NIMV) employed as the first-line respiratory support. When NIMV failed or respiratory distress worsened, invasive mechanical ventilation was initiated using volume-controlled assist-control (V-ACV) mode. Tidal volume was set at 4–6 mL/kg of ideal body weight. In all patients, invasive ventilation was initiated with a baseline PEEP of 5 cmH_2_O. Thereafter, FiO_2_ and PEEP levels were titrated individually to achieve optimal oxygenation based on the patient’s response. Positive end-expiratory pressure (PEEP) was optimized individually to ensure adequate oxygenation. To minimize the risk of barotrauma, maximum airway pressure (Pmax) was generally kept below 35 cmH_2_O, though transient increases up to 40 cmH_2_O were permitted in severe cases. Sedation was guided by hemodynamic status: ketamine was preferred in hypotensive patients, while propofol and fentanyl were used in normotensive individuals. In cases of ventilator asynchrony or refractory hypoxemia, continuous infusion of rocuronium was used to achieve neuromuscular blockade.

### 2.5. Statistical Analysis

Statistical analyses were conducted using IBM SPSS Statistics version 27.0 (IBM Corp., Armonk, NY, USA). The normality of continuous variables was assessed using the Shapiro–Wilk test. Continuous variables were expressed as medians with interquartile ranges (IQRs) or means with standard deviations (SDs), depending on data distribution. Categorical variables were reported as frequencies and percentages. Comparisons between the COVID-19 and non-COVID-19 groups were performed using the Mann–Whitney U test for non-normally distributed continuous variables and the Student’s *t*-test for normally distributed variables. Categorical variables were compared using the chi-square test. A *p*-value of <0.05 was considered statistically significant. Multivariable logistic regression analysis was performed to identify factors independently associated with mortality. Variables with *p* < 0.05 in univariate analysis were included in the multivariate model. Multicollinearity was assessed, and variables with a correlation coefficient > 0.75 were excluded. Model fit was evaluated using the Omnibus test and was found to be significant (*p* < 0.01). A *p*-value of less than 0.05 was considered statistically significant.

## 3. Results

### 3.1. Study Populations

**Figure 1:**
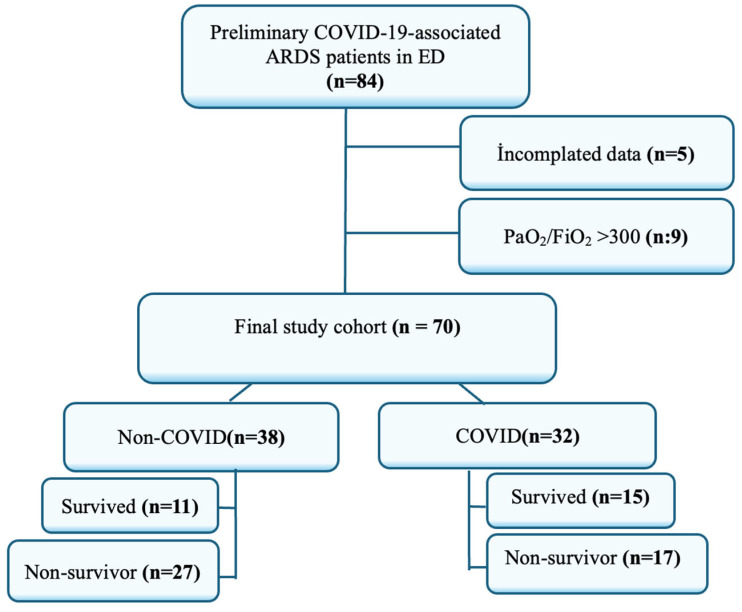
Flow Chart of Participants.

A total of 84 patients who were admitted to the emergency department (ED) with a preliminary diagnosis of COVID-19-associated ARDS were initially evaluated. Of these, 5 patients were excluded due to incomplete data, and 9 were excluded because their PaO_2_/FiO_2_ ratio was greater than 300. As a result, 70 patients were included in the final study cohort. Among the included patients, 38 were classified as non-COVID-related ARDS and 32 as COVID-related ARDS. In the non-COVID-19 group, 11 patients survived and 27 did not. In the COVID-19 group, 15 patients survived while 17 did not (Figure 1).

### 3.2. Patient Demographics and Comorbidities

Baseline demographic characteristics and comorbidities did not differ significantly between the COVID-19 and non-COVID-19 groups. The median age was 77.5 years (IQR: 61.3–86.0) in the COVID-19 group and 74.5 years (IQR: 66.5–82.5) in the non-COVID-19 group (*p* = 0.65). The proportion of male patients was similar (59.4% vs. 57.9%, *p* = 0.90). Although most comorbidities were evenly distributed, congestive heart failure was more common in the non-COVID-19 group, with no significance (63.2% vs. 40.6%, *p* = 0.06; Table 1). Charlson comorbidity index (CCI) did not differ between the two groups (*p* = 0.42).

### 3.3. Laboratory Parameters

Laboratory parameters were largely comparable between the COVID-19 and non-COVID-19 groups. There were no statistically significant differences in white blood cell count, hemoglobin, creatinine, or C-reactive protein (CRP) levels. Platelet count was found to be higher in the COVID-19 group, with no significance (219 × 10^3^/µL vs. 186 × 10^3^/µL, *p* = 0.06; Table 2).

### 3.4. Mechanical Ventilator Parameters and Mortality

Among the ventilatory parameters, plateau pressure, driving pressure, and PEEP values were significantly higher in the COVID-19 group compared to the non-COVID-19 group (*p* = 0.01). There were no significant differences between the groups in terms of static compliance, tidal volume, or mechanical power (MP) (Table 3).

Ventilator parameters and Charlson Comorbidity Index (CCI) were compared between survivors and non-survivors. Mechanical power, driving pressure, plateau pressure, Pmax, and PEEP were significantly higher in the non-survivor group compared to the survivor group (*p* = 0.01). There were no significant differences between the groups in terms of static compliance or tidal volume. The median CCI value was significantly higher in the non-survivor group than in the survivor group (6 [5–8] vs. 5.5 [4–6], *p* = 0.02) (Table 4).

### 3.5. Clinical Scoring Systems

Among clinical scoring systems, the SOFA score was significantly higher in the COVID-19 group compared to the non-COVID-19 group (3.5 vs. 3.0, *p* = 0.02). The APACHE II and PSI scores did not show significant differences between groups. The mortality rates were recorded as 71.1% in the non-COVID-19 group and 53.1% in the COVID-19 group; but the difference was not significant (*p* = 0.12; Table 5).

In the COVID-19 group, non-survivors exhibited significantly higher APACHE II scores compared to survivors (30.7 ± 7.66 vs. 23.7 ± 8.60, *p* = 0.02). Although median PSI and SOFA scores were numerically higher in non-survivors (142 vs. 119 and 4 vs. 3, respectively), these differences were not statistically significant (*p* = 0.11 and *p* = 0.88, respectively). Similarly, there was no significant difference in the P/F ratio between the groups (81.4 [57.4–165] vs. 102 [85.5–177], *p* = 0.27) (Table 6).

### 3.6. Logistic Regression Analysis of Mortality

Multivariate logistic regression analysis was performed to identify variables independently associated with in-hospital mortality. The model included three variables: mechanical power, Charlson comorbidity index and COVID-19 test results (positive vs. negative). The model fit was confirmed using the omnibus test (*p* < 0.01), which indicated that the model was statistically significant. The model explained approximately 80% of the variance in mortality (Nagelkerke R^2^ = 0.804).

According to the Likelihood Ratio (LR) analysis, MP contributed the most to the model, while CSI and the COVID-19 test result provided similar levels of contribution (LR test χ^2^ values were 53.89 (*p* < 0.001), 5.84 (*p* = 0.033), and 5.05 (*p* = 0.041), respectively). The results of the logistic regression analysis are presented in Table 7.

**Figure 2:**
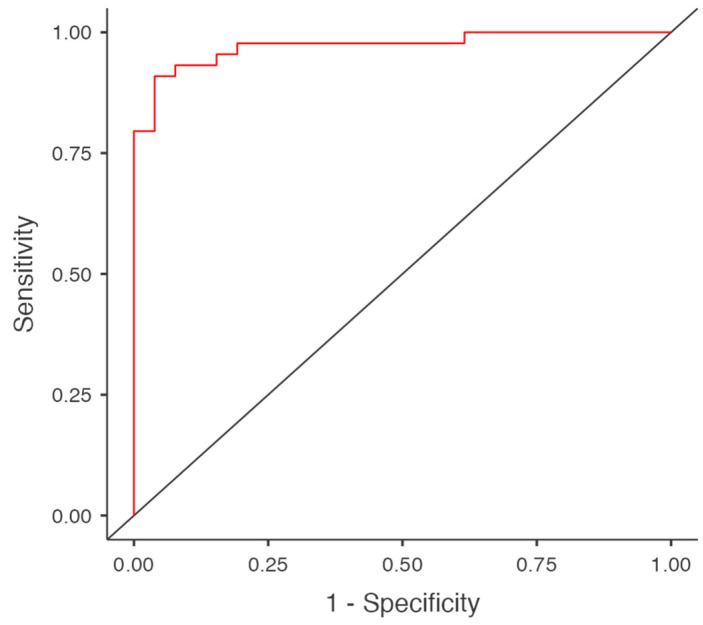
ROC Curve of the Model.

The diagnostic accuracy of the model was assessed using the area under the curve (AUC) of the receiver operating characteristic (ROC) curve, which was calculated as 0.972. The sensitivity and specificity were 84.6% and 95.5%, respectively. The model’s accuracy was 91%, indicating good predictive power (Figure 2).

## 4. Discussion

Acute respiratory distress syndrome (ARDS) remains a major challenge in the management of critically ill patients, with the emergence of COVID-19 adding further complexity to its clinical presentation and ventilatory management. Although COVID-19-associated ARDS shares many features with classical ARDS, ongoing debate persists regarding whether it represents a distinct physiological entity requiring tailored ventilatory strategies [4]. In this study, we sought to examine and compare the early respiratory mechanics and outcomes of patients with COVID-19 and non-COVID-19 ARDS managed in the emergency department. By analyzing data from the initial phase of invasive ventilation, we aimed to identify early mechanical ventilation characteristics that might help inform future research and clinical decision-making in this setting.

Demographic characteristics and comorbidity profiles in our cohort appeared largely comparable between COVID-19 and non-COVID-19 ARDS patients. This finding aligns with Bain et al., who reported similar age distributions but a higher body mass index (BMI) in COVID-19 patients [5]. In contrast, Brault et al. observed that COVID-19 patients were generally older and had a higher prevalence of obesity and diabetes, while immunosuppression was more frequent among non-COVID-19 cases [7]. These discrepancies may be attributed to differences in study populations, geographic settings, and temporal context.

Our findings are consistent with and contribute to previous studies examining the prognostic value of early ventilatory mechanics in ARDS. In our cohort, mechanical power (MP) values measured within the first 120 min were observed to be higher among non-survivors (21.6 vs. 16.8 J/min, *p* = 0.01), and this early association was statistically significant in our multivariate model (OR: 3.33; 95% CI: 1.18–6.33). However, as MP was recorded at a single early time point, this association should be interpreted cautiously. It may reflect an initial physiological burden rather than a sustained prognostic indicator, especially considering that downstream ventilatory strategies and cumulative exposure were not captured in our dataset. This observation is consistent with the findings of Azizi et al. [8], who reported that higher mechanical power within the first 24 h was associated with increased 30-day mortality. However, their study included serial measurements and ICU-level data, whereas our findings are limited to an early ED snapshot. Thus, while the direction of association is similar, its prognostic implications in our setting remain exploratory. Static compliance did not significantly differ between survivors and non-survivors, supporting the findings of Vandenbunder et al. [9], who reported that reduced compliance in COVID-19 ARDS lacked prognostic significance. In contrast, Boscolo et al. [10] identified a compliance threshold (<48 mL/cmH_2_O) and driving pressure > 14 cmH_2_O as predictors of mortality. Although driving pressure was significantly higher in our non-survivor group (16.5 vs. 14.5 cmH_2_O, *p* = 0.01), it did not remain significant in multivariate analysis. Collectively, these results may suggest that while individual ventilatory parameters—such as plateau pressure, driving pressure, or compliance—may reflect respiratory compromise, mechanical power, as a composite indicator reflecting multiple ventilatory pressures and volumes, may provide additional insight into the initial physiological burden in ARDS. However, its role as a reliable predictor of mortality cannot be firmly established based on a single early measurement, particularly without longitudinal ventilatory data. Nevertheless, the higher Charlson Comorbidity Index observed among non-survivors (median: 6 vs. 5.5, *p* = 0.02) indicates that underlying comorbidity burden may have also contributed to outcomes, potentially diminishing the predictive utility of isolated respiratory mechanics.

The observed differences in respiratory mechanics, such as reduced compliance and elevated plateau pressures in COVID-19 ARDS patients, may be partially attributed to the distinct pathophysiologic mechanisms of the disease—namely, diffuse alveolar damage, endothelial injury, and microvascular thrombosis—as outlined by Ball et al. [11].

The limited prognostic value of early respiratory mechanics observed in our cohort is consistent with prior studies suggesting that static parameters alone may not fully capture the complexity of COVID-19–related respiratory failure. Li Bassi et al. reported that early respiratory system compliance was not predictive of ICU mortality but was modestly associated with earlier ICU discharge, highlighting its limited utility as a standalone prognostic marker [12]. Similarly, Pan et al. demonstrated that despite elevated driving pressures and low compliance in COVID-19 ARDS patients, the majority were poorly recruitable as determined by a low recruitment-to-inflation ratio, suggesting that conventional mechanical indices may poorly reflect underlying lung pathophysiology and recruitability [13]. Adding to this paradigm, Rovas et al. provided compelling evidence that microvascular dysfunction—quantified via sublingual microcirculation imaging—and glycocalyx degradation strongly correlate with disease severity and organ dysfunction in sepsis [14]. Together, these studies and our findings suggest that ventilator mechanics should not be interpreted in isolation but rather within the broader context of systemic inflammation, endothelial injury, and microvascular failure, which likely contribute significantly to outcomes in ARDS, particularly in COVID-19–related cases. In line with this perspective, Pelosi et al. advocate for a personalized ventilation approach in ARDS, integrating physiological parameters, imaging data, and evolving patient characteristics rather than relying solely on static mechanical indices [15].

Gutiérrez et al. demonstrated that increased driving pressure (≥12 cmH_2_O) and ventilatory ratio (≥2) measured at 72 h were independently associated with mortality [16]. While we similarly observed higher driving pressure in non-survivors within the first 120 min of invasive mechanical ventilation (16.5 vs. 14.5 cmH_2_O, *p* = 0.01), this parameter did not retain statistical significance in multivariable analysis, possibly due to the shorter observation window. Martínez et al. [17]. found no significant association between initial static compliance and 28-day mortality in COVID-19 patients, which is consistent with our data showing comparable compliance between survivors and non-survivors (23.5 vs. 24.9 mL/cmH_2_O). Lastly, Maamar et al. [18]. reported no significant difference in 60-day mortality between COVID-19 and influenza ARDS despite some early mechanical differences—paralleling our finding that COVID-19 and non-COVID-19 patients had similar mortality rates (*p* = 0.12). Importantly, mechanical power measured within 120 min was significantly higher in non-survivors (21.6 vs. 16.8 J/min, *p* = 0.01) and independently associated with in-hospital mortality. However, this association was based on short-term measurements, and whether mechanical power measured during early ED ventilation retains prognostic significance throughout the ICU course remains unclear and warrants further investigation with serial measurements and broader outcome data.

Puah et al. found that COVID-19 ARDS patients with initially high lung compliance who experienced a significant decline in compliance over time had worse outcomes, with a mortality rate of 33.3% compared to 11.6% in the low compliance group [19]. While this appears counterintuitive, it highlights the dynamic nature of lung injury progression. In our study, static compliance measured within the first 120 min did not differ significantly between survivors and non-survivors and was not associated with mortality. Similarly, Panwar et al. showed in a pre-COVID-19 ARDS cohort that although low compliance was associated with higher mortality, no clear threshold could define risk [20]. Mohanty et al. provided a pathophysiological explanation for this dissociation, describing diffuse alveolar damage and microvascular thrombosis in COVID-19 lungs—lesions that may not be reflected in early mechanical parameters [21]. These observations may support the notion that early compliance alone may not reliably predict outcome and that temporal evolution and vascular pathology play a critical role in disease severity.

Li Bassi et al. reported that rising creatinine and worsening PaO_2_/FiO_2_ ratios were significant predictors of 28-day mortality in mechanically ventilated COVID-19 patients [22]. In our cohort, although creatinine levels at presentation did not differ significantly between groups, the COVID-19 group exhibited more severe hypoxemia, with a significantly lower P/F ratio compared to non-COVID-19 patients (98.4 vs. 136 mmHg, *p* = 0.02). Despite this, mortality rates between the groups were similar, suggesting that isolated hypoxemia may not be sufficient to predict outcome in the absence of progressive organ dysfunction.

In our study, the SOFA score was significantly higher in COVID-19 patients compared to non-COVID-19 patients; however, no clinical scoring system effectively distinguished survivors from non-survivors when analyzed across the entire cohort. This partial divergence aligns with the findings of Citu et al., who reported that both SOFA and qSOFA scores were significantly associated with in-hospital mortality in COVID-19 patients, though their practical use may vary with clinical setting [23]. Similarly, while PSI did not differ significantly between survivors and non-survivors in our cohort, Chen et al. found that PSI and CURB-65 were reliable for identifying low-risk patients, whereas APACHE II showed higher discriminatory value for mortality [24]. Mehryar et al. also observed significantly elevated APACHE II scores among deceased COVID-19 patients, though they concluded that its overall predictive accuracy was limited [25]. Taken together, these studies and our findings suggest that although classical scores like SOFA and APACHE II may reflect severity trends in COVID-19, their standalone use for early outcome prediction—especially in mixed ARDS populations—may be insufficient without integrating organ-specific and dynamic clinical data.

Zou et al. demonstrated that both APACHE II and SOFA scores were strong independent predictors of ICU mortality in COVID-19 patients, with APACHE II showing excellent discriminatory performance (AUC: 0.966) [26]. In line with this, our subgroup analysis of COVID-19 patients revealed that non-survivors had significantly higher APACHE II scores compared to survivors (30.7 vs. 23.7, *p* = 0.02). However, SOFA and PSI scores did not significantly differ between outcome groups, echoing the findings of Onuk et al., who reported no prognostic value for these scores despite markedly elevated cytokine levels among non-survivors [27]. Beigmohammadi et al. similarly found that while both SOFA and APACHE II scores were higher in non-survivors, SOFA had superior predictive accuracy in their ICU cohort [28]. These collective findings suggest that although traditional scoring systems may reflect illness severity at presentation, their mortality prediction capacity in COVID-19 ARDS varies depending on context and may benefit from integration with dynamic or biomarker-based assessments.

Higgins et al. demonstrated that ICU patients with COVID-19 had significantly higher standardized mortality ratios and length of stay compared to non-COVID-19 viral pneumonia cohorts, suggesting greater illness severity and ICU resource burden despite risk adjustment [29]. In our study, while overall in-hospital mortality did not significantly differ between COVID-19 and non-COVID-19 ARDS patients, APACHE II scores were significantly higher among non-survivors in the COVID-19 subgroup, reflecting greater disease burden in line with Higgins’ observations. Similarly, Yang et al. reported that SOFA scores were highly predictive of both clinical severity and mortality in COVID-19 patients [30]. Although SOFA scores were also higher in our COVID-19 cohort, they did not differentiate survivors from non-survivors, possibly due to differences in timing of assessment, patient heterogeneity, or early intervention at the emergency department level. These findings may indicate that while physiologic scoring systems like APACHE II and SOFA can capture severity trends, their predictive accuracy may be influenced by patient setting and timing of evaluation.

In addition to classical ventilator parameters, recent literature emphasizes the importance of incorporating comorbidity and physiological scores in mortality prediction models. Romanelli et al. demonstrated that high CCI values significantly increased the risk of non-invasive ventilation failure in critically ill COVID-19 patients, highlighting the relevance of comorbidity burden in respiratory management [31]. Moreover, in a broader ICU population, Romanelli et al. utilized machine learning techniques to combine CCI with SOFA and SAPS II scores for cluster-based risk stratification. Their findings showed that these clustered models significantly improved mortality prediction compared to non-clustered approaches [32]. These results support the need for multidimensional approaches that go beyond ventilator mechanics alone when estimating prognosis in ARDS patients.

### Limitations

The present study has several limitations that should be considered when interpreting the findings. First, it was conducted at a single academic center and retrospectively, which may limit the generalizability of the results to other settings with different patient populations and resource availability. Second, ventilator parameters and clinical scores were recorded at a single time point—within the first 120 min of mechanical ventilation—during the emergency department phase, potentially omitting dynamic changes in respiratory mechanics or severity scores that could occur in the subsequent ICU course. Third, although patients were managed with standardized ventilators, individual clinician-driven adjustments may have introduced variability in settings such as tidal volume or PEEP. Although the relatively small sample size—particularly when divided by PCR status and survival outcomes—may have reduced the ability to detect subtle differences in some variables, this study remains one of the few that highlights how emergency physicians manage critically ill patients. Additionally, data on the exact timing of intubation or duration of prior non-invasive ventilation were not consistently available in our dataset, which limits our ability to assess the impact of delayed invasive ventilation on outcomes. The interpretation of mechanical power as a prognostic marker is limited by the fact that it was recorded only once during early ED ventilation. Longitudinal exposure, changes during ICU care, and cumulative ventilatory stress were not assessed, which restricts conclusions regarding its long-term prognostic relevance. Lastly, we were unable to evaluate short-term emergency department–relevant outcomes such as vasopressor requirement, oxygenation response, or early clinical deterioration, due to the retrospective nature of data collection. These proximal endpoints may have better reflected the impact of early ventilatory mechanics and should be prioritized in future prospective studies.

## 5. Conclusions

In this cohort of ARDS patients managed with mechanical ventilation in the emergency department, COVID-19 cases exhibited higher plateau pressures, PEEP, and driving pressures compared to non-COVID-19 patients. Early ventilatory parameters such as compliance and tidal volume did not show significant associations with in-hospital mortality. Among clinical severity scores, SOFA was significantly higher in the COVID-19 group compared to non-COVID-19 patients, while APACHE II did not differ between groups overall but was significantly elevated among non-survivors within the COVID-19 subgroup.

Additionally, mechanical power and driving pressure were higher in non-survivors across the entire cohort, and the Charlson Comorbidity Index was also significantly elevated, indicating that both ventilatory burden and baseline comorbidities may contribute to worse outcomes. These findings underscore the multifactorial nature of ARDS mortality and the limitations of relying solely on early respiratory mechanics for prognostic assessment.

## Figures and Tables

**Table 1:** Baseline characteristics of study groups.

Demographic Characteristics and Comorbidities	Non-COVID (n = 38)	COVID-19 (n = 32)	*p*-Value
Age (Median, IQR 25–75)	74.5 (66.5–82.5)	77.5 (61.3–86.0)	0.65
Male Gender n, (%)	22 (57.9%)	19 (59.4%)	0.90
CHF n, (%)	24 (63.2%)	13 (40.6%)	0.06
MAP (mmHg)	81.7 (69.3–93.2)	73.3 (63.3–97.5)	0.77
CKD/AKI n, (%)	11 (28.9%)	8 (25.0%)	0.71
CVD n, (%)	7 (18.4%)	3 (9.7%)	0.61
DM n, (%)	19 (50.0%)	15 (46.9%)	0.79
CAD n, (%)	14 (36.8%)	9 (28.1%)	0.43
COPD n, (%)	13 (34.2%)	17 (53.1%)	0.11
Neuropsychiatric Disorders n, (%)	5 (13.2%)	5 (15.7%)	1.00
Malignancy n, (%)	6 (15.8%)	3 (9.4%)	0.49
CCI (Median, IQR 25–75)	6 (5–7)	6 (3.75–7)	0.42

The Chi-square test and Fisher’s exact test were used for statistical analysis. Abbreviations: CHF: Congestive Heart Failure; CKD: Chronic Kidney Disease; AKI: Acute Kidney Injury; CVD: Cerebrovascular Disease; DM: Diabetes Mellitus; CAD: Coronary Artery Disease; COPD: Chronic Obstructive Pulmonary Disease; MAP: Mean Arterial Pressure; CCI: Charlson Comorbidity index.

**Table 2:** Comparison of laboratory parameters by group.

Parameter	Non-COVID (Median, IQR 25–75)	COVID-19 (Median, IQR 25–75)	*p*-Value
WBC (×10^3^/µL)	11.2 (8.15–17.8)	12.0 (8.22–18.5)	0.39
Hemoglobin (Hgb, g/dL)	12.7 (11.0–14.1)	13.3 (10.0–14.1)	0.86
Hematocrit (Hct, %)	39.2 (34.4–43.9)	38.2 (31.9–44.1)	0.49
Platelet Count (Plt, ×10^3^/µL)	186 (155–265)	219 (193–321)	0.06
Urea (mg/dL)	82.0 (42.3–114)	62.0 (44.3–102)	0.63
BUN (mg/dL)	38.5 (18.8–52.8)	38.0 (18.8–57.0)	0.86
Creatinine (mg/dL)	1.33 (0.95–1.53)	1.25 (0.98–1.71)	0.68
Sodium (mmol/L)	139 (137–142)	139 (136–143)	0.99
Potassium (mmol/L)	4.55 (3.92–5.07)	4.30 (3.88–4.90)	0.55
Glucose (mg/dL)	161 (123–221)	150 (119–232)	0.80
AST (IU/L)	27.5 (18.0–67.3)	35.0 (20.0–81.3)	0.42
ALT (IU/L)	21.5 (15.3–38.3)	23.0 (17.8–44.5)	0.36
CRP (mg/dL)	70.5 (17.3–148)	106 (39.8–176)	0.36

Abbreviations: WBC: White Blood Cell Count; Plt: Platelet Count; Hgb: Hemoglobin Level; Hct: Hematocrit Level; BUN: Blood Urea Nitrogen; AST: Aspartate Transaminase; ALT: Alanine Transaminase; CRP: C-reactive Protein.

**Table 3:** Comparison of ventilator parameters by group.

Parameter	Non-COVID (Median, IQR 25–75)	COVID-19 (Median, IQR 25–75)	*p*-Value
Compliance (Crs, mL/cm H_2_O)	24.9 (22.5–27.9)	23.3 (21.1–26.8)	0.12
Tidal Volume (mL)	400 (381–420)	415 (400–421)	0.20
PEEP (cm H_2_O)	10 (7–12)	12 (10–14)	0.01 *
Pmax (cm H_2_O)	35 (35–38)	35 (35–40)	0.25
Pplat (cm H_2_O)	25 (21–27)	30 (26–30)	0.01 *
ΔPrs (cm H_2_O)	15.00 (13.3–17.0)	16.50 (15.8–18.0)	0.01 *
MP	20.2 (16.9–21.9)	19.8 (17.8–22.5)	0.80

*: Significant (*p* < 0.05); PEEP: Positive End-Expiratory Pressure; Pmax: Maximum Pressure; Pplat: Plateau Pressure; ΔPrs: Driving Pressure; MP: Mechanical Power.

**Table 4:** Comparison of ventilator parameters by mortality status.

Parameter	Survivors (Median, IQR 25–75)	Non-Survivors (Median, IQR 25–75)	*p*-Value
Compliance (Crs, mL/cm H_2_O)	24.9 (22.5–29.6)	23.5 (21.4–26.4)	0.14
Tidal Volume (mL)	400 (400–420)	400 (394–450)	0.53
PEEP (cm H_2_O)	9 (6–10)	12 (10–12.5)	0.01 *
Pmax (cm H_2_O)	35 (32–35)	38 (35–40)	0.01 *
Pplat (cm H_2_O)	24 (20.3–26)	28 (25–30)	0.01 *
ΔPrs (cm H_2_O)	14.5 (13.3–16.0)	16.5 (15.0–18.0)	0.01 *
MP	16.8 (15.4–17.9)	21.6 (20.0–23.5)	0.01 *
CCI (Median, IQR 25–75)	5.50 (4–6)	6 (5–8)	0.02

*: Significant (*p* < 0.05); Mann–Whitney U test was performed. PEEP: Positive End-Expiratory Pressure; Pmax: Maximum Pressure; Pplat: Plateau Pressure; ΔPrs: Driving Pressure; MP: Mechanical Power CCI: Charlson Comorbidity index.

**Table 5:** Comparison of Clinical Scores and Outcomes by Group.

Parameter		Non-COVID	COVID-19	*p*-Value
APACHE-2 (±SD)		25.3 ± 7.20	27.4 ± 8.75	0.27
PSI (IQR 25–75)		116 (96–144)	137.5 (93–147.2)	0.34
SOFA (IQR 25–75)		3 (3–3)	3.5 (3.0–4.0)	0.02
P/F ratio		136 (105–166)	98.4 (63.8–168)	0.02
Mortality	Survivors	11 (28.9%)	15 (46.9%)	0.12
	Non-Survivors	27 (71.1%)	17 (53.1%)	

APACHE-2: Acute Physiology and Chronic Health Evaluation-2 Score; SOFA: Sequential Organ Failure Assessment Score; PSI: Pneumonia Severity Index.; P/F: PaO_2_/FiO_2_.

**Table 6:** Comparison of clinical scores by mortality in COVID-19 patients.

Parameter	Survivors (n:15)	Non-Survivors (n:17)	* p*-Value
APACHE-2 (±SD)	23.67 ± 8.60	30.71 ± 7.66	0.02
PSI (IQR 25–75)	119 (79.5–143)	142 (125–151)	0.11
SOFA (IQR 25–75)	3 (3–4)	4 (3–4)	0.88
P/F ratio IQR 25–75	102 (85.5–177)	81.4 (57.4–165)	0.27

APACHE-2: Acute Physiology and Chronic Health Evaluation-2 Score; SOFA: Sequential Organ Failure Assessment Score; PSI: Pneumonia Severity Index.; P/F: PaO_2_/FiO_2_.

**Table 7:** Multivariate logistic regression analysis of mortality.

Variables	Estimate (b)	Wald	*p*-Value	Odds Ratio (OR)	OR %95 CI
Intercept	−23.916	−3.57	<0.001	4.11 × 10^−11^	8.02 × 10^−17^–2.10 × 10^−5^
MP	1.203	3.67	<0.001	3.33	1.175–6.326
CCI	0.441	2.13	0.033	1.55	1.037–2.331
COVID-19 Results (positive-negative)	−2.137	−2.04	0.041	0.12	0.015–0.921

MP: Mechanical Power; CCI: Charlson Comorbidity index.

## Data Availability

The datasets used and/or analyzed during the current study are available from the corresponding author upon reasonable request.
